# Neighborhood History-based Social Walking for Caregivers and People Living with Early-Stage Dementia

**DOI:** 10.1093/geroni/igaf122.3321

**Published:** 2025-12-31

**Authors:** Patrice Fuller, Charles Fennell, Nora Mattek, Michael Crespo, Raina Croff

**Affiliations:** Oregon Health & Science University, Portland, Oregon, United States; Wake Forest University School of Medicine, Winston-Salem, North Carolina, United States; Oregon Health & Science University, Portland, Oregon, United States; Oregon Health & Science University, Portland, Oregon, United States; Oregon Health & Science University, Portland, Oregon, United States

## Abstract

The SHARP Caregiver study engaged seven triads (n = 21) consisting of a healthy or mildly cognitively impaired (MCI) caregiver (age >40), their care partner with MCI or early-stage dementia, (age >40), and a healthy or MCI support person (age >18) in Black history-based neighborhood walking. Triads used the SHARP application to walk 1-mile routes with images to prompt conversational reminiscence 3x/week over 16 weeks in Portland, Oregon’s gentrifying historically Black neighborhoods. Mean age overall was 69.8 years. Eight (38%) were cognitively healthy [mean MoCA 26.5 (SD 1.9)] and 13 (4 MCI; 9 early-stage dementia) were cognitively impaired [mean MoCA 18.8 (SD 2.8)]. Caregivers and care partners completed pre/post health assessments. Overall, 29% rated themselves very/fairly active at baseline and 57% at end-study; 36% rated themselves in excellent/very good health at baseline and 43% at end-study. Pre/post mean Geriatric Depression Scale scores decreased for caregivers from 1.86 to 1.14 and increased for care partners from 1.40 to 1.80, indicating normal/no depression and consistency over time. Pre/post mean Zarit Burden Interview (short 12-item) scores for caregivers increased from 13.14 to 14.86 indicating mild to moderate burden and consistency over time. Highly motivational program aspects were recording memories for use in community learning sessions about healthy aging and being accountable to their group. Overall, self-rated activity level improved while depression and caregiver burden was maintained at healthy levels. The SHARP-Caregiver study is feasible for caregivers and care partners, providing structure to weekly activity, group accountability and socializing for mental health and community connection.

